# Supplementary material for: Patient self-reported pain and nausea via smartphone following day care surgery, first year results: An observational cohort study
Source: PLOS Digit Health. 2024 Jul 10;3(7):e0000342. doi: 10.1371/journal.pdig.0000342 (PMC11236166; doi:10.1371/journal.pdig.0000342)
Supplement: S1 Table — (DOCX) [file pdig.0000342.s001.docx]

| **Overview surgical specialism and most proceeded interventions** | **Total cohort** | **Active** | **Not active** | **Not downloaded** |
| --- | --- | --- | --- | --- |
| Cases, n (%) | 4952 | 351 (7%) | 241 (5%) | 4360 (88%) |
| **Surgical specialism**  Anaesthesia  Ear nose throat  Eye  Gynaecology  Neuro  Oral / maxillofacial  Orthopaedic  Plastic  General / Trauma  Urology | 27 (1%)  582 (12%)  62 (1%)  506 (10%)  19 (<1%)  61 (1%)  724 (15%)  389 (8%)  2541 (51%)  40 (1%) | 1 (<1%)  31 (9%)  1 (<1%)  38 (11%  3 (1%)  5 (1%)  41 (11%)  18 (5%)  212 (61%)  0 | 0  37 (15%)  2 (<1%)  23 (10%)  0  4 (2%)  24 (10%)  20 (8%)  131 (54%)  0 | 26 (1%)  514 (12%)  59 (1%)  445 (10%)  16 (<1%)  52 (1%)  659 (15%)  351 (8%)  2198 (50%)  40 (1%) |
| **Surgical intervention***  Partial excision of breast tissue  Laparoscopic gastric bypass  Implantation of internal device into ankle joint  Open reduction of wrist joint  Tonsillectomy  Laparoscopic cholecystectomy  Open reduction of carpal or metacarpal  Repair of inguinal hernia  Local excision of lesion uterus  Implantation of device into bone of forearm  Open reduction of bone of shoulder  Endoscopic paranasal sinusectomy  Diagnostic exploratory laparascopy  Septoplasty  Partial ostectomy, tarsal or metatarsal | 237 (5%)  224 (5%)  209 (4%)  188 (4%)  177 (4%)  160 (3%)  146 (3%)  132 (3%)  128 (3%)  124 (3%)  116 (2%)  115 (2%)  109 (2%)  100 (2%)  90 (2%) | 19 (5%)  34 (10%)  24 (7%)  4 (1%)  12 (3%)  11 (3%)  0  7 (2%)  10 (3%)  32 (10%)  5 (1%)  3 (1%)  9 (3%)  6 (2%)  2 (1%) | 17 (7%)  16 (7%)  11 (5%)  4 (2%)  17 (7%)  9 (4%)  6 (3%)  5 (2%)  3 (1%)  10 (4%)  5 (2%)  6 (3%)  2 (1%)  6 (3%)  2 (1%) | 201 (5%)  174 (4%)  174 (4%)  180 (4%)  148 (3%)  140 (3%)  140 (3%)  120 (3%)  115 (3%)  82 (2%)  106 (2%)  106 (2%)  98 (2%)  88 (2%)  86 (2%) |

**S1 Table. Overview surgical specialism and most proceeded interventions**

*15 (out of 197) most proceeded surgical interventions named and coded according to the World Health Organisation (WHO) international classification of health interventions (ICHI); n = number
