# Supplementary material for: Patient self-reported pain and nausea via smartphone following day care surgery, first year results: An observational cohort study
Source: PLOS Digit Health. 2024 Jul 10;3(7):e0000342. doi: 10.1371/journal.pdig.0000342 (PMC11236166; doi:10.1371/journal.pdig.0000342)
Supplement: S1 STROBE Checklist — (DOC) [file pdig.0000342.s002.doc]

STROBE Statement: Monitoring of Self-Recorded Pain and Nausea via Smartphone Following Day Care Surgery, First Year Results: An Observational Cohort Study

|  | Item No | Recommendation |
| --- | --- | --- |
| **Title and abstract** | 1 | (*a*) … An Observational Cohort Study |
|  |  | (*b*) yes |
| Introduction | | |
| Background/rationale | 2 | ...Remote monitoring with a direct feedback loop between patients and healthcare professionals to tailor pain and nausea management could overcome these problems and improve clinical patient outcomes… |
| Objectives | 3 | The present study aimed to contribute to the evidence of mobile health by evaluating the self-recorded postoperative pain and nausea scores of patients using the remote monitoring app one year after its implementation in day care. In addition, we evaluated the uptake and actual use of the app by the patients to assess whether the routine provision of such a remote monitoring tool is feasible |
| Methods | | |
| Study design | 4 | From 10/02/2020 to 29/03/2021, a prospective observational cohort study was conducted in OLVG Hospital, a large teaching hospital with two locations in Amsterdam, the Netherlands |
| Setting | 5 | Yes |
| Participants | 6 | (*a*) *Cohort study*— The following inclusion criteria were required: age >18 years and scheduled for day-care surgery. The exclusion criterion was unplanned stay in the hospital after day care surgery. |
| Variables | 7 | yes |
| Data sources/ measurement | 8* | yes |
| Bias | 9 | Not applicable, only descriptive analysis |
| Study size | 10 | yes |
| Quantitative variables | 11 | yes |
| Statistical methods | 12 | (*a*) yes |
|  |  | (*b*) yes |
|  |  | (*c*) yes |
|  |  | (*d*) *Cohort study*—not applicable |
|  |  | (*e*) Describe any sensitivity analyses |

Continued on next page

| Results | | |
| --- | --- | --- |
| Participants | 13* | (a) yes |
|  |  | (b) yes |
|  |  | (c) yes |
| Descriptive data | 14* | (a) yes |
|  |  | (b) yes |
|  |  | (c) yes |
| Outcome data | 15* | *Cohort study*—yes |
|  |  |  |
|  |  |  |
| Main results | 16 | (*a*) yes |
|  |  | (*b*) not applicable |
|  |  | (*c*) not applicable |
| Other analyses | 17 | Not applicable |
| Discussion | | |
| Key results | 18 | Yes |
| Limitations | 19 | Yes |
| Interpretation | 20 | Yes |
| Generalisability | 21 | Yes |
| Other information | | |
| Funding | 22 | yes |

*Give information separately for cases and controls in case-control studies and, if applicable, for exposed and unexposed groups in cohort and cross-sectional studies.

**Note:** An Explanation and Elaboration article discusses each checklist item and gives methodological background and published examples of transparent reporting. The STROBE checklist is best used in conjunction with this article (freely available on the Web sites of PLoS Medicine at http://www.plosmedicine.org/, Annals of Internal Medicine at http://www.annals.org/, and Epidemiology at http://www.epidem.com/). Information on the STROBE Initiative is available at www.strobe-statement.org.
