# Supplementary material for: Patient self-reported pain and nausea via smartphone following day care surgery, first year results: An observational cohort study
Source: PLOS Digit Health. 2024 Jul 10;3(7):e0000342. doi: 10.1371/journal.pdig.0000342 (PMC11236166; doi:10.1371/journal.pdig.0000342)
Supplement: Attachment — Submitted filename: Response to the Reviewers.docx [file pdig.0000342.s003.docx]

Response to Reviewers

Date: 6-11-2023

Submission: PDIG-D-23-00297

Title: Patient self-reported pain and nausea via smartphone following day care surgery, first year results: An observational cohort study PLOS Digital Health

Dear Editorial Board, dear Haleh Ayatollahi,

On behalf of all authors who contributed to this manuscript, I thank you for the opportunity to submit a revised version. We are grateful to the section editor and the reviewers for their valuable comments and suggestions for improvement. Below we address each comment separately.

**Editorial comments:**

1. In the Funding Information you indicated that no funding was received. Please revise the Funding Information field to reflect funding received.

*Funding information is revised.*

2. Please provide separate figure files in .tif or .eps format only and remove any figures embedded in your manuscript file. Please also ensure that all files are under our size limit of 10MB.

*Figure files are removed from the manuscript file and provided as separate file in .tif format.*

3. We do not publish any copyright or trademark symbols that usually accompany proprietary names, eg ©, ®, ™ (e.g. next to drug or reagent names). Please remove all instances of trademark/copyright symbols throughout the text, including ® on page 5

*All instances of trademark/copyright symbols are removed throughout the text, including ® on page 5*

**Reviewer comments:**

Reviewer #1: The manuscript of Thiel et al describes a retrospective study of patients self-reported pain and nausea after daycare surgery using a smartphone application. Digital solutions have the potential to improve the interactions between medical professionals and their patients. The present study evaluated the adoption rate and post-operative complications (pain, nausea) of patients by self-reported ratings. Overall, the results are of great interest for the growing community of digital health professionals. The manuscript could be improved by clarifying the issues stated below.

*Reviewer #1, we wish to express our gratitude for your recognition of the significance of this manuscript. Below we will address all your questions and comments to the best of our abilities.*

Major issues

1) This is not the first app that was developed to monitor and/or report post-operative complications. Please include a paragraph where you compare your app to other common alternatives.

1a) State the advantages (e.g. language, data integration, unique features) and disadvantages of your software when compared to the existing alternatives.

*Thank you for this suggestion.*

*We have included a section in the METHODS, smartphone application:*

*The OLVG pain app was developed in collaboration between patients (including patient council), healthcare professionals, the Dutch Society of Anesthesiology and Logicapps. As was commissioned by the Department of Anaesthesiology of OLVG Hospital. The usability of the application was evaluated in a proof of concept study amongst 50 patients and 12 stakeholders (e.g. anaesthesiologists, patients from patient council) [1]. Both patients and stakeholders agreed that the application was easy to use, and its simplicity and design were well suited for pain recording. Furthermore, patients were willing and motivated to use the application for recording their pain. The difference in median pain intensity scores between those recorded by patients using the app and those recorded by nurses was not statistically significant [1]. Two important recommendations from the proof of concept study have been implemented in the current version; the ability to also capture nausea as a patient-reported outcome and the direct transfer of the entered data to the electronic patient record.*

*We have included the following sections in the DISCUSSION:*

*Despite the promising results of our previous proof-of-concept study [1], the uptake and use of the application was surprisingly low. This is contrast with the results of the SATELIA application [2] that is the most comparable app with direct electronic record integration, and pain and side effect monitoring. Notably, it achieved a high rate of active users on POD1, potentially due to proactive nurse follow-up. We recognize this from our own results that active motivation of patients by one of the researchers present in the day care ward during the last 3 months of the follow-up period has resulted in 285 out of 351 patients actively engaging with the application.*

*In brief, to compare of the OLVG pain app with other recently published results of applications designed for post-operative follow-up [2-5]. The Mserv application [3] is an Android-exclusive app for thoracic and urogenital procedures, active within hospital setting. In contrast, the OLVG pain app that is accessible to outpatients and clinically admitted patients and is compatible with both iOS and Android operating systems. RecoverWell application [4], which was studied in a breast cancer surgery trial, offers advantages such as photo follow-up and drain monitoring but lacks the crucial feature of integration with electronic medical records, a feature that the OLVG pain app has. The Q1.6 Inguinal Hernia app [5] uses 'twitch crowdsourcing' for real-time data collection but does not provide patient follow-up. In addition to these, there are other recent applications that focus more on rehabilitation and revalidation for specific surgical procedures, featuring built-in patient instructions and reminders rather than primarily focusing on the management of postoperative pain and nausea [6-8].*

1b) The frequency of patients that were in pain or feeling nauseous and nothing was done is quite high in this study (see Figure 2 and Figure 3). In the beginning it is stated that the use of an smartphone application could be advantageous for improving patient care.

2) Please discuss possible reasons for this observation. Are changes to the implementation necessary and if so which ones would be most beneficial for the patient?

Consider including an analysis of patient satisfaction in the subgroups where something was done and nothing was done.

*Figures 2 and 3, as well as Table 2, depict the outcomes of the in-app questions.*

*We may not have presented our observed results clearly enough. For instance, on POD1, out of 229 active users, 174 (76%) reported experiencing pain. Additionally, 30 (9%) of the patients reported pain and described it as unbearable, while 27 (8%) of them reported pain, considered it unbearable, and indicated a desire for some form of relief. It is important to emphasize that these outcomes solely represent the patient's responses to the in-app questions and do not confirm whether they actually received any additional attention or care.*

*We have included the following is section to Secondary outcome: It is important to emphasize that these outcomes solely represent the patient's responses to the in-app questions and do not confirm whether they actually received any additional attention or care*

*For clarification, we have modified Figure 2 and adjusted the legend*

Incidence of postoperative pain

Patients

| Postoperative day | The number of patients active with the app | The number of patients recording pain, regardless of their NRS score or valuation | The number of patients recording unbearable pain and a request for relief |
| --- | --- | --- | --- |
| POD 1 | 229 | 174 | 27 |
| POD 2 | 182 | 112 | 12 |
| POD 3 | 160 | 105 | 4 |
| POD 4 | 135 | 79 | 3 |
| POD 5 | 131 | 70 | 1 |
| POD 6 | 108 | 56 | 2 |
| POD 7 | 100 | 44 | 4 |

*For clarification, we have modified Figure 3 and adjusted the legend*

Incidence of postoperative nausea

Patients

| Postoperative day | The number of patients active with the app | The number of patients recording nausea, regardless of their valuation | The number of patients recording nausea and a request for relief |
| --- | --- | --- | --- |
| POD 1 | 229 | 63 | 6 |
| POD 2 | 182 | 48 | 4 |
| POD 3 | 160 | 31 | 2 |
| POD 4 | 135 | 21 | 0 |
| POD 5 | 131 | 20 | 1 |
| POD 6 | 108 | 18 | 2 |
| POD 7 | 100 | 12 | 0 |

Minor issues

line 98: Smartphone requirements, ability to understand the Dutch language and cognitive impairment (e.g. dementia) are likely to lead to lower adoption to the proposed digital application. However, those patients were not excluded in the present study. The limitations should state that these factors were not assessed and could lead to a reduction in the adoption rate

*We agree that this is an addition regarding the limitations.*

*The following is added to the Limitations: The ability to understand the Dutch language and cognitive impairment (e.g., dementia) is likely to lead to lower adoption of the proposed digital application. These factors were not assessed and recorded, and they might result in reduced adoption. However, it's important to note that patients with mental impairments or those prone to delirium, do not undergo day care surgery in our practice."*

line 102: Was there a specific reason that the researcher was only present for the last 3 months of the observation period? And did the presence of the researcher lead to time-dependent differences of active users during this period?

*The specific reason for the researcher's presence was the Covid-19 restrictions, which required social distancing. Researchers were only permitted to visit the day care ward during the last three months of the study observation period. This led to a time-dependent difference during the period the researcher was present 285 out of 351 patients were engaging with the application.*

*We have added the following to the discussion*

*We recognize this from our own results that active motivation of patients by one of the researchers present in the day care ward during the last 3 months of the follow-up period has resulted in 285 out of 351 patients actively engaging with the application.*

*In relation to this, the presence of a researcher on the day care ward, who could offer additional guidance to the patients about the application and provide instructions on its usage, was only allowed during the last 3 months of the study's observation.*

Table 1: The subtitle of the table should be re-written. I would recommend that you use superscript symbols (e.g.†,¶,…) behind p-values in the table. In the the annotation of the table you can write the corresponding test for each symbol. The current style is confusing for the reader.

Furthermore, in the methods section it is written that median and IQR are used for non-normal distributed variables. In the table median and range is used. Please decide what strategy you want to use.

In the SES row the commata should probably be substitutes with dots

*All comments and remarks are addressed as suggested.*

line 223: Consider changing the statement „None of the patients experienced any severe pain.“ to „Retrospectively, none of the patients reported that they experienced any severe pain.“. In line 213 it is mentioned that 14% reported NRS scores above 7, so either all those patients dropped out or more likely they did not remember their pain as vividly.

*Thank you for your suggestion*

*We have added the following to the RESULTS, Overall experienced pain, nausea, satisfaction with the application and received assistance:*

*Reflecting on the initial 7 days post-surgery, none of the patients who completed the in-app closeout questions indicated the presence of severe pain.*

line 233: The 11% is in contrast to the 12% mentioned earlier.

*This has been corrected.*

Reviewer #2: The conclusion of the study presented in this retrospective cohort investigation reflects a comprehensive evaluation of remote monitoring through a specialized smartphone application (the OLVG Pain app) in the context of pain and postoperative nausea and vomiting (PONV) management after outpatient surgery. The analysis took place one year following the app's integration into routine clinical practice. The primary findings unveiled a rather modest adoption rate, as only 11% of eligible patients actually downloaded the app, and merely 7% of the total patient pool engaged with it for pain and nausea reporting. Among active users, most symptom reports occurred within the initial two days post-discharge.

The study's identification of the highest proportion of pain reports on postoperative days (POD)1 and 2, with up to 14% indicating severe pain, aligns with existing literature. Notably, the prevalence of moderate-to-severe pain reported in this study, at 14% on POD 1, differs from earlier research showing higher rates, potentially due to variances in pain assessment thresholds and the patient demographics under examination.

Regarding PONV, the research identifies a relatively elevated occurrence of nausea among patients, yet a small percentage sought relief. Comparable studies in the realm of PONV after ambulatory surgery have demonstrated similar incidences, though methodological disparities and the study's relatively low response rate hinder direct comparison. The sole study assessing PONV up to POD 7 exhibited a 6% nausea occurrence rate on that day, signifying the need for more comprehensive investigations.

Despite the encouraging outcomes of a prior proof-of-concept study, the actual implementation and utilization of the application proved surprisingly subdued. Anticipations of greater post-discharge usage were not met, prompting consideration of Rogers's diffusion of innovation theory, which suggests that current users are early adopters and innovators, and broader adoption is yet to be achieved. A potentially underestimated factor, the digital divide, comes to the forefront as a potential constraint, particularly given the socioeconomic background of OLVG Hospital patients, primarily residing in economically disadvantaged Amsterdam neighborhoods. This raises concerns about equitable access and capability to utilize e-health solutions, particularly among the less privileged.

The research acknowledges its limitations, inherent to observational cohort studies, and highlights the ongoing impact of the COVID-19 pandemic on study outcomes, given its influence on surgical scheduling and patient demographics. The advantages of self-reported outcomes, untainted by healthcare professional influence, are noted. However, the limited user engagement and sporadic symptom reporting impose constraints on result interpretation. Future inquiries should delve into the acceptance and effectiveness of mobile health applications for remote monitoring in the context of recovery.

In conclusion, the study offers valuable insights into the implementation and outcomes of remote monitoring via a specialized smartphone application for pain and PONV management post-outpatient surgery. The investigation underscores the challenges of adoption and the potential influence of socioeconomic factors, in addition to recognizing the impact of ongoing global events. These findings emphasize the significance of continued research to elucidate the broader implications and benefits of mobile health solutions in optimizing patient care.

*Reviewer #2: We would like to express our gratitude to the reviewer for his insightful analysis of the results we presented. Policymakers and hospital managers have placed their hopes on e-health applications to make healthcare more efficient and cost-effective. However, it remains uncertain to what extent e-health can contribute to this goal regarding our results. Particularly, the uptake of our application has been extremely disappointing. On the one hand, it appears that the majority of the current patient population lags behind in terms of innovation. On the other hand, there may be a digital divide, where patients from neighbourhoods with a low socio-economic status have less access to e-health services. With this manuscript, we aim to convey a message to the readers of Plos Digital Health, especially those involved in implementing health apps in daily practice, to be mindful of the aforementioned factors when they also experience a disappointing uptake.*

Reviewer #3: The paper aims to show the results of a one year study of post-operative patients using a pain reporting app.

*Reviewer 3#, Thank you for reviewing our paper. Below we will address all your questions and comments to the best of our abilities.*

1) I cannot access the supplemental files --

*The supplemental files have been resubmitted*

2) As a matter of style, please start sentences with letters and avoid starting with numbers (eg, "56% of patients...")

*This is addressed as suggested*

3) Line 263: there is mention of first generation and non-Western types of patients but there is no direct study data to support this - these were linked to the SES demographic data (summarized) rather than to the specific individual data. Suggest to remove these observations.

*The following is adjusted in the manuscript: This is supported by the results of a recent study conducted in Amsterdam among mothers from low-SES backgrounds, which shows that poverty and the complexity of information and communication technology, influence perspectives, experiences, and ICT-related needs [9].*

4) Were patient feedback on the usability of the app obtained? If yes, that will be an important statement. If patient feedback were not, then that could be stated briefly as well.

*This is now addressed in the manuscript by referring to a previously conducted proof of concept study with the application: The usability of the application was researched in a proof of concept study amongst 50 patients and 12 stakeholders (e.g. anaesthesiologists, patients from patient council) [1]. Both patients and stakeholders agreed that the application was easy to use, and its simplicity and design were well suited for pain recording. Furthermore, patients were willing and motivated to use the application for recording their pain.*

*Moreover, it was already stated in the present manuscript that 60 patients completed the in-app close out questions on POD7 most of these patients 57 (95%) were satisfied with the app and reported that it was okay, pleasant, or very pleasant. A minority of 3 (5%) patients reported that it was not pleasant or unpleasant. The received assistance was rated as very pleasant, pleasant, or Ok by 56 (93%) patients, while 4 (7%) patients rated it as not pleasant or very unpleasant.*

5) Line 129: mismatch between "a patient" and "they" -- pls match the plurality

*This has been corrected.*

6) Patients were given medication (minor) to take home. Did the app document if the patient took them regularly? Or just that a patient intervention (generic) was done?

*In the application, it is not possible to record the prescribed and used medication. The research data only includes the medication a patient was prescribed upon discharge. We agree with the reviewer that recording medication data could be an important addition to the functionality of the application. Moreover, this feature is integrated in the version of the application currently used at OLVG Hospital.*

7) Of the patients who were on oxycodone, were these directly observed by a health professional prior to discharge or were these self-administered? Pls mention as this could have an impact on the pain scores.

*Pain and nausea medication was self-administered by the patient at home. As stated now in the manuscript they received written and verbal instructions how to manage postoperative pain and nausea.*

The paper can contribute significantly on the knowledge about mobile app uptake of patients. Suggest referencing either the UTAUT and Delone-Mclean frameworks to identify which aspects of technology adoption were covered by the study and which were not.

*We thank the reviewer for this valuable feedback and the recommendation that our paper can contribute to the knowledge about mobile app uptake by patients. It demonstrates insight into e-health innovation by taking a meta-level perspective on the application's implementation process. Indeed, we previously conducted such an analysis in a prior study using the model of Faber et al.*

*With due respect to the reviewer's suggestions, we prefer to refer to the e-Health adoption model of Faber et. al. 2017 [10] and our own research project on eHealth adoption conducted in OLVG Hospital [11].*

*The following has been added to the manuscript: We anticipated that more patients used the application after discharge. We had identified and addressed important implementation themes from a meta viewpoint namely; size of the hospital, top management support, organizational readiness, centralization in decision- making and absorptive capacity [10, 12]. We found that the most important factor influencing the adoption of the OLVG pain app was absorptive capacity, primarily determined by the innovation’s urgency, relevance, and a sense of ownership and responsibility. We addressed this by; enhancing the innovation's relevance, involving stakeholders from the outset, and assigning ambassadors and managers to support stakeholder engagement and to provide proper guidance and training [11].*

Reviewer #4: Thank you for the opportunity to review this manuscript entitled “Patient self-reported pain and nausea via smartphone following day care surgery, first year results: An observational cohort study”.

There has certainly been a shift towards elective daycase surgery which provides an attractive cohort of patients that would potentially benefit from digital health interventions.

Unfortunately, however I do not believe the article provides sufficient methodological or clinical benefit that I would be able to recommend it for publication in PLOS Digital Health, even in the case of substantial revision. Further justification for this decision is outlined below:

The included primary outcome was the incidence of patients with self-reported pain and nausea in the early post-operative period – however this doesn’t really provide much information about the clinical utility of the app, i.e. ultimately we want to how the app influences treatment compared to standard practice. This information is present in very small numbers in terms of those that have pain/nausea and have had something done, but there is no way to compare with traditional pathways, and the numbers included are insufficient to draw any meaningful conclusions.

There was limited information included as to the types of clinical cases performed, which would likely have a significant impact on the perceived rates of pain and nausea. For example, general surgical patients would likely have significant differences in the subsequent levels of nausea compared to orthopaedic patients. Heterogeneity of this population makes it very difficult to draw any useful information regarding rates for more specific sub-populations where clinical teams are likely to be working.

There was a quite dramatic lack of uptake for the number of patients included in the study. This again limits the potential generalisability of study findings given such a small sub-sample responded, and that there is likely to be significant bias within this population of responders. It would likely be far more useful to an audience to try in much greater depth to identify why the uptake was so poor. This would likely provide greater reach of utility, for example providing potential key information to those current going through the process of app development or similar attempts at clinical practice integration.

*Reviewer #4, Thank you for reviewing and commenting our manuscript. We regret that you are unable to recommend our manuscript for publication in Plos Digital Health. Hoping to counter your arguments, the following: we still think that the present manuscript is of relevance for the readers of Plos Digital Health. Not primarily because of the outcomes regarding postoperative pain and nausea, but more importantly to provide insight the disappointing uptake and factors that may have contributed to this as we addressed in the discussion of the manuscript. However, we agree that we have to be careful making conclusions about the clinical utility of the application and that evaluating the clinical utility of such an application and its potential benefits for patients requires a different research design. We have, initiated a randomized controlled trial, the protocol of which has been published [13]. The primary objective of this trial is to assess the difference in perceived quality of recovery after day care surgery between patients with or without a smartphone application for remote monitoring, as measured by the QoR-15. If interested, we are willing to share the results of our still ongoing RCT when they are analysed.*

References

1.	Thiel, B., et al., *Patient reported postoperative pain with a smartphone application: A proof of concept.* PLoS One, 2020. **15**(5): p. e0232082.

2.	Carlier, J., et al., *Pain evaluation after day-surgery using a mobile phone application.* Anaesth Crit Care Pain Med, 2021. **40**(3): p. 100879.

3.	Gordon-Williams, R., et al., *An Interactive Pain Application (MServ) Improves Postoperative Pain Management.* Pain Res Manag, 2021. **2021**: p. 8898170.

4.	Temple-Oberle, C., et al., *Effect of Smartphone App Postoperative Home Monitoring After Oncologic Surgery on Quality of Recovery: A Randomized Clinical Trial.* JAMA Surg, 2023. **158**(7): p. 693-699.

5.	van Hout, L., W.J.V. Bokkerink, and P. Vriens, *Clinical feasibility of the Q1.6 Inguinal Hernia application: a prospective cohort study.* Hernia, 2023. **27**(2): p. 449-458.

6.	Kasnakova, P., et al., *Randomized controlled trial of multidisciplinary rehabilitation therapy using mobile applications in cases of ankle fractures.* Eur J Transl Myol, 2022. **32**(2).

7.	Yang, C., et al., *Remote Follow-up with a Mobile Application Is Equal to Traditional Outpatient Follow-up After Bariatric Surgery: the BELLA Pilot Trial.* Obes Surg, 2023. **33**(6): p. 1702-1709.

8.	Yu, C., et al., *Smartphone-based application to improve medication adherence in patients after surgical coronary revascularization.* Am Heart J, 2020. **228**: p. 17-26.

9.	Goedhart, N.S., et al., *‘Just having a computer doesn’t make sense’: The digital divide from the perspective of mothers with a low socio-economic position.* New Media & Society, 2019. **21**(11-12): p. 2347-2365.

10.	Faber, S., M. van Geenhuizen, and M. de Reuver, *eHealth adoption factors in medical hospitals: A focus on the Netherlands.* Int J Med Inform, 2017. **100**: p. 77-89.

11.	Thiel, B., et al., *Adoption of a Postoperative Pain Self-Report Tool: Qualitative Study.* JMIR Hum Factors, 2022. **9**(2): p. e33706.

12.	Greenhalgh, T. and C. Papoutsi, *Spreading and scaling up innovation and improvement.* BMJ, 2019. **365**: p. l2068.

13.	Thiel, B., et al., *Quality of recovery after day care surgery with app-controlled remote monitoring: study protocol for a randomized controlled trial.* Trials, 2023. **24**(1): p. 102.
