# Supplementary material for: Patient self-reported pain and nausea via smartphone following day care surgery, first year results: An observational cohort study
Source: PLOS Digit Health. 2024 Jul 10;3(7):e0000342. doi: 10.1371/journal.pdig.0000342 (PMC11236166; doi:10.1371/journal.pdig.0000342)
Supplement: Attachment — Submitted filename: Response to the ReviewersII.docx [file pdig.0000342.s004.docx]

Response to Reviewers

Date: 22-12-2023

Submission: PDIG-D-23-00297R1

Title: Patient self-reported pain and nausea via smartphone following day care surgery, first year results: An observational cohort study PLOS Digital Health

Dear Editorial Board, dear Haleh Ayatollahi,

On behalf of all authors who contributed to this manuscript, I thank you for the opportunity to submit a revised version. We are grateful to the section editor and the reviewers for their valuable comments and suggestions for improvement. Below we address each comment separately.

**Additional Editor Comments (if provided):**

**I appreciate the authors for thier time and efforts to revise the manuscript. Please address the following comments in your next revision, too.**

**1- Please add appropriate keywords after the abstract.**

*We added the following keywords after the abstract: Day care surgery, Postoperative, Self Recording, Smartphone application, Uptake, Innovation adoption*

**2- In the introduction section, please review other similar applications that have been developed so far.**

*Thank you for your comment; however, we feel that this has already been adequately addressed in the current version of the manuscript in response to the questions of reviewer#1.*

*The following sections are already in the DISCUSSION: Despite the promising results of our previous proof-of-concept study [1], the uptake and use of the application was surprisingly low. This is contrast with the results of the SATELIA application [2] that is the most comparable app with direct electronic record integration, and pain and side effect monitoring. Notably, it achieved a high rate of active users on POD1, potentially due to proactive nurse follow-up. We recognize this from our own results that active motivation of patients by one of the researchers present in the day care ward during the last 3 months of the follow-up period has resulted in 285 out of 351 patients actively engaging with the application.*

*In brief, to compare of the OLVG pain app with other recently published results of applications designed for post-operative follow-up [2-5]. The Mserv application [3] is an Android-exclusive app for thoracic and urogenital procedures, active within hospital setting. In contrast, the OLVG pain app that is accessible to outpatients and clinically admitted patients and is compatible with both iOS and Android operating systems. RecoverWell application [4], which was studied in a breast cancer surgery trial, offers advantages such as photo follow-up and drain monitoring but lacks the crucial feature of integration with electronic medical records, a feature that the OLVG pain app has. The Q1.6 Inguinal Hernia app [5] uses 'twitch crowdsourcing' for real-time data collection but does not provide patient follow-up. In addition to these, there are other recent applications that focus more on rehabilitation and revalidation for specific surgical procedures, featuring built-in patient instructions and reminders rather than primarily focusing on the management of postoperative pain and nausea [6-8].*

**3- Some figures of the application should be added to the manuscript.**

*The following figures are added to the manuscript, the numbering of the consecutive figures has been corrected:*

**Figure 1**

**Figure 2**

**Figure 3**

**Reviewers' comments:**

*We thank reviewer #3 and reviewer #5 for their work on our manuscript and their positive response to the changes we made in the previous revision. We do not see questions or remarks requiring attention at this time.*

**References**

1.	Thiel, B., et al., *Patient reported postoperative pain with a smartphone application: A proof of concept.* PLoS One, 2020. **15**(5): p. e0232082.

2.	Carlier, J., et al., *Pain evaluation after day-surgery using a mobile phone application.* Anaesth Crit Care Pain Med, 2021. **40**(3): p. 100879.

3.	Gordon-Williams, R., et al., *An Interactive Pain Application (MServ) Improves Postoperative Pain Management.* Pain Res Manag, 2021. **2021**: p. 8898170.

4.	Temple-Oberle, C., et al., *Effect of Smartphone App Postoperative Home Monitoring After Oncologic Surgery on Quality of Recovery: A Randomized Clinical Trial.* JAMA Surg, 2023. **158**(7): p. 693-699.

5.	van Hout, L., W.J.V. Bokkerink, and P. Vriens, *Clinical feasibility of the Q1.6 Inguinal Hernia application: a prospective cohort study.* Hernia, 2023. **27**(2): p. 449-458.

6.	Kasnakova, P., et al., *Randomized controlled trial of multidisciplinary rehabilitation therapy using mobile applications in cases of ankle fractures.* Eur J Transl Myol, 2022. **32**(2).

7.	Yang, C., et al., *Remote Follow-up with a Mobile Application Is Equal to Traditional Outpatient Follow-up After Bariatric Surgery: the BELLA Pilot Trial.* Obes Surg, 2023. **33**(6): p. 1702-1709.

8.	Yu, C., et al., *Smartphone-based application to improve medication adherence in patients after surgical coronary revascularization.* Am Heart J, 2020. **228**: p. 17-26.
