# Supplementary material for: Patient self-reported pain and nausea via smartphone following day care surgery, first year results: An observational cohort study
Source: PLOS Digit Health. 2024 Jul 10;3(7):e0000342. doi: 10.1371/journal.pdig.0000342 (PMC11236166; doi:10.1371/journal.pdig.0000342)
Supplement: Attachment — Submitted filename: Response to Reviewers III.docx [file pdig.0000342.s005.docx]

Response to Reviewers

Date 26/2/2024

PDIG-D-23-00297R2

Title: Patient self-reported pain and nausea via smartphone following day care surgery, first year results: An observational cohort study

Dear Editorial Board, dear Haleh Ayatollahi,

On behalf of all authors who contributed to this manuscript, I thank you for the opportunity to submit a revised version. We are grateful to the editor and the reviewer for their valuable comments and suggestions for improvement. Below we address each comment separately.

**Additional Editor Comments (if provided): -**

**Reviewers' comments:**

Reviewer #6: This paper provides a description of a smartphone app allowing day surgery patients to self-report post-operative pain and nausea, and receive health professional intervention for distressing levels of symptoms. The authors found a relatively low uptake (7% of the study population became active users of the app), but among those found that pain and nausea were most common on post-op days 1 and 2, and fell off quickly afterwards. This is an important contribution to the growing field of smartphone apps for healthcare interaction. The utility of the app is demonstrated, and the paper lays groundwork for future studies regarding the role of patient preferences in leveraging digital tools for streamlined access to the healthcare system, and likely provides a baseline for future interventional studies aimed at modifying pain and nausea symptoms after discharge.

*We would like to express our gratitude to Reviewer #6 for working on our manuscript, as well as for the positive response to the changes in the previous revision. Furthermore, we appreciate the positive statement on the added value of our results to the field of research on smartphone applications for health interaction.*

*In the following, we will answer and address the additional comments:*

Reviewer #6: Raw data is not shared, citing GDPR. Although there are many institutional and legal barriers to sharing healthcare data, data sharing is critical to promote reproducible research. Could the authors share a de-identified repository of their raw data?

*We agree that data sharing is critical to promote reproducible research. However, as already mentioned, due to institutional and legal constraints we are unable to share our data publicly. The data will be stored for 15 years on a secured server at OLVG Hospital. For Interested researchers it is always possible to access the data by submitting an application to the local research advisory committee of OLVG Hospital, referencing the study with number WO 20.239 at www.acwo@olvg.nlBovenkant formulier*

Reviewer #6: The authors suggest low socioeconomic status contributed to low uptake of the smartphone app. This is certainly possible. However, in the current digital climate many consumers have concerns about data privacy and the value-added of downloading additional apps to their phone. Do the authors feel that these “savvy consumer” concerns may have impacted uptake as well? What was the data privacy policy associated with the app (e.g., was the data transmitted or maintained via any commercial 3rd party?), and were prospective patients counseled on this? Should consideration be given to alternate digital formats, e.g. a standard website vs an app, or text message communication to collect the same data?

*In this comment, there are four questions that we will answer separately:*

*We acknowledge the possibility that concerns about data privacy and the reluctance to download additional apps might have influenced app uptake. However, we have no data on patient concerns about privacy and downloading additional applications, so we cannot draw conclusions on this topic.*

*The application is institution-specific built for OLVG, ensuring transfer of data directly to the patient's electronic health record through a secure FHIR HL-7 server connection without the interference of a commercial third party.*

*During the preoperative assessment, patients were informed about the purpose of the application, and a summary with the instructions was sent by email or letter, as already mentioned in the manuscript.*

*We have no data on the patients' preferences to use alternate digital formats. Indeed, this could be an interesting topic for future research to evaluate.*

Reviewer #6 Please explain what is meant by “the patient council” in line 135

*We appreciate the reviewer’s request for clarification. The patient council is an advisory board of patients affiliated with the hospital. They voluntarily provide advice and approval on organizational changes as well as on healthcare innovation and scientific research.*

Reviewer #6 Line 166-167 – should this say “with the help of a software specialist”?

*This is corrected*

Reviewer #6 Line 195 – suggest “injury-related” rather than “traumatic” surgeries

*This is corrected*

Reviewer 6# Table 2 – for the bottom four rows, please clarify (in caption or table) if values represent the number of people who answered the question at all on a particular day, or the number with an affirmative response.

*We appreciate the reviewer’s request for clarification. We have added the following in caption: For the variables; Are you in pain?; Not bearable?; Request to relief the pain; Are you nauseous?; and Request to relief nausea: n (%) represents the number and percentage of patients answering the question with an affirmative response.*

Reviewer #6 Line 218 – is the median times used overall or per day?

*The median times used is overall. We have corrected this in the manuscript with the following: In total, 351 unique patients were active users during the 7 days postoperative follow period, with an overall median times used of 2 (range, 1–8).*

Reviewer 6# There are several areas of the paper that require copy editing for standard English, in particular lines 69 and 70 (suggest present tense verbs), 96 (ordering of month/date is different for American and European audiences, suggest just writing out the month and date), 157 (primary outcome was the incidence), 160-164 (these are list items and are not full sentences, suggest separate with semicolon), 222 (“not bearable and requested relief”), 223 (non-bearable vs no bearable?), 230 (clarify), 233 (extra “.”), 264 (“would use” the app), 280 (“average resident”)

*We thank the reviewer for these corrections that contribute to the clarity of the manuscript and improve the spelling for standard English. They have all been addressed appropriately in the revised version of the manuscript.*

*On behalf of all authors*

*Yours sincerely,*

*Bram Thiel*
